# Bioinert Fibrous Polypropylene Membranes via In Situ Polymerization of Zwitterionic Poly(sulfobetaine methacrylate)

**DOI:** 10.1021/acs.langmuir.4c04226

**Published:** 2025-02-10

**Authors:** Gian Vincent Canlas Dizon, Chiao-Ling Chang, Chih-Chen Yeh, Chung-Jung Chou, Jheng-Fong Jhong, Jie Zheng, Yung Chang

**Affiliations:** † R&D Center for Membrane Technology and Department of Chemical Engineering, Chung Yuan Christian University, 200 Chung Pei Rd, Taoyuan 32023, Taiwan; ‡ PuriBlood Medical, Baoshan, Hsinchu 300096, Taiwan; § Department of Chemical, Biomolecular, and Corrosion Engineering, The University of Akron, Akron, Ohio 44325, United States

## Abstract

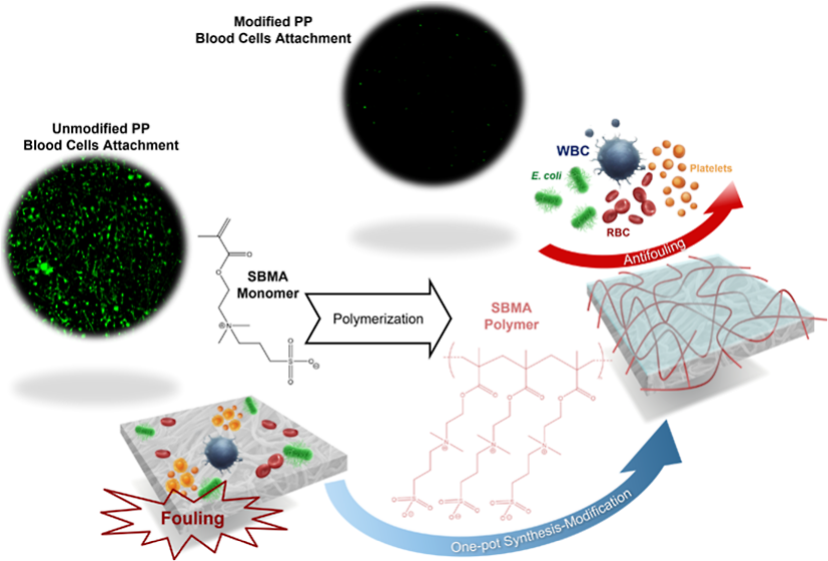

This study reports the fabrication of a biocompatible polypropylene (PP) fibrous membrane via an in situ polymerization process, generating a dual network of PP fibers and poly(sulfobetaine methacrylate) (poly(SBMA)). In this method, the synthesis of the polymer and the modification process happen in a single step. Notably, the modification was achieved without the incorporation of hydrophobic groups in the modifying polymer, demonstrating that the physical entanglement of poly(SBMA) and PP was sufficient to produce a stable biocompatible membrane. The presence of the poly(SBMA) coating was confirmed through various characterization techniques. A reduction in the water contact angle indicated increased hydrophilicity, while Fourier-transform infrared spectroscopy and X-ray photoelectron spectroscopy analyses verified the presence of poly(SBMA) on the PP membrane surface. The PP membranes were modified with varying sulfobetaine methacrylate solid. The physical morphology of the modified membranes was observed via SEM, and it was seen that membranes modified with higher solid content (4.00, 7.50, 15.0, and 30.0 wt %) showed significant polymer aggregates, making the membranes significantly denser than the original PP membrane. Therefore, optimal modification was achieved with 1.00 wt % poly(SBMA), which balanced enhanced hydrophilicity with preservation of the structural integrity of the membrane. This modification resulted in a 70% reduction in bacterial (*Escherichia coli*) attachment and a 60% reduction in blood cell attachment compared to the unmodified PP membrane.

## Introduction

Fouling is a common problem for a lot of materials. This phenomenon occurs when something attaches on the surface of a material and the function of the material is affected negatively. Specifically, for biomaterials, biofouling happens when bacteria, cells, or other biomolecules attach on their surface. The adhesion of microorganisms or other biological foulants to material surfaces is primarily influenced by factors, such as surface energy, surface charge, surface chemistry, and hydrophobicity. These criteria were formulated based on minimizing protein adsorption, which could be considered the onset of biofouling. On the contrary, it was shown that electrically neutral and hydrophilic surfaces are relatively less prone to biofouling^1−3^ because they can form a strong and stable hydration layer that prevents attachment of other biomolecules. Biofouling could lead to many complications such as infection, inflammation, or ultimately implant rejection by our body. For instance, in artificial heart valves or vascular stents, the accumulation of biofoulants can lead to thrombus formation and subsequent device malfunction, not only reducing their therapeutic efficacy but also posing a risk of death.^4,5^ To overcome these challenges, researchers have been devoted to developing surface modification techniques that could impart resistance to biofouling. These techniques include altering the surface wettability of materials via surface coatings or incorporating micro- or nanopatterns on the surface.^6−8^

Zwitterionic materials have emerged as a promising class of antifouling agents due to their ability to resist nonspecific protein adsorption, a critical factor in preventing biofouling. This property was notably demonstrated by Whitesides and co-workers, who showed that self-assembled monolayers (SAMs) of zwitterionic molecules could significantly reduce nonspecific protein adsorption.^9^ To date, there are many different zwitterionic chemistries used in antifouling applications. However, noteworthy structures include carboxybetaine methacrylate (CBMA),^10^ 2-methacryloyloxyethyl phosphorylcholine (MPC),^11^ and sulfobetaine methacrylate (SBMA).^12^ Each of these zwitterionic materials exhibits excellent antifouling properties, though they also possess unique characteristics. For instance, Professor Jiang’s group highlighted the functionalization potential of CBMA due to its carboxylic acid groups.^13^ MPC, on the other hand, offers broader solubility compared to other zwitterionic chemistries,^14^ while SBMA is notable for its ease of synthesis.^15^ Despite SBMA being more accessible, one challenge associated with its use is the solubility of its polymeric form. Solubility issues can complicate surface modification processes, as conventional techniques typically require the modifying material to be dissolved in a solvent. This limitation presents a potential drawback when SBMA is incorporated in surface modification applications.

Surface modification techniques can be generally classified into three methods: surface grafting, surface coating, and blending.^16−18^ Surface grafting involves the growth of the modifying polymer on the surface of the material, while surface coating involves using already formed polymers usually containing a group having interaction with the material, letting the polymer anchor on the surface of the material.^19−23^ Blending, on the other hand, involves the addition of the polymeric additive to the bulk of the material before its formation into a surface. This method is more common with the modification of polymeric membranes such as polyvinylidene fluoride where the dope solution before phase transition takes place.^24^ Recently, an in situ self-assembly process has emerged as an alternative approach to address the solubility limitations of polymeric sulfobetaine-based copolymers.^25,26^ This method involves the simultaneous polymerization and immobilization of SBMA and styrene in the presence of azobis(isobutyronitrile) and the target material. Through optimization of the process, including control of the molecular weight and monomer ratios, the copolymer is anchored to the material surface via the hydrophobic styrene segments. However, this approach requires careful optimization of multiple parameters, including monomer ratios and reaction conditions, to achieve an optimal performance.

In this study, polypropylene (PP) fibrous membranes will be modified similarly to the in situ polymerization process but only using SBMA. With only SBMA in the reaction solution, this will lead to the formation of a dual network of polySBMA and the PP fibrous membrane. The formation of this dual network would lead to a simpler and more stable zwitterionization procedure for porous or fibrous materials. Physical and chemical characterizations were performed to give evidence to the modification process. Lastly, the antifouling performance of the membranes were also assessed with bacteria and human whole blood. The hydrophilicity, coating density, morphology, and other chemical properties of the membranes were correlated with their antifouling performance.

## Experimental Section

### Materials

The meltblown PP fibrous membrane used in this study was provided by Mytrex Industries, Incorporated. The monomer used in the modification process, SBMA, was obtained from Sigma-Aldrich. Ammonium peroxodisulfate (APS) served as the initiator, and *N*,*N*,*N*′,*N*′-tetramethylethylenediamine (TEMED) as the catalyst, both purchased from Echo Chemical Co. HPLC grade methanol was purchased from Uni-Onward Corporation. Deionized water was acquired using a PURELAB Chorus 1 ELGA purification system, producing deionized water with a resistivity of 18.2 MΩ cm. Whole blood was provided from healthy individuals and used as is.

### Methods

#### Interpenetrating Zwitterionic Surface Modification of a Polypropylene Fibrous Membrane

The SBMA monomer was dissolved in deionized water and methanol with a volume ratio of 3:7 and stirred until completely homogeneous. The solution was then bubbled with nitrogen to reduce dissolved oxygen from the solvents, which may affect the reaction. The PP membrane was then placed inside the bottle. A stock solution of APS (in DI water) and TEMED (in methanol) having a concentration of 5 mg/mL was freshly prepared. Then, corresponding amounts of the APS and TEMED solutions were added to the bottle. The reaction was performed in various solute concentrations as shown in Table 1. The reaction was then sealed and placed in an oil bath maintained at 60 °C for 24 h while constantly stirring with a magnetic stir bar. After the reaction, the PP membrane was retrieved from the bottle and then washed three times by ultrasonication in deionized water and methanol in order to remove any unreacted or weakly attached coating. Finally, the PP membranes were then placed in a 50 °C oven for 1 h and transferred to a freeze drier until fully dry.

**Table 1:** Amount of Each Component in the Reaction of PolySBMA

solid content (wt %)	amount of solutes			volume of solvent (mL)	
	SBMA (g)	APS (μL)	TEMED (μL)	DI water	methanol
0.05	0.0122	10	6	7.383	21.711
0.10	0.0245	20	20	7.370	21.686
1.00	0.2448	200	200	7.123	21.310
4.00	1.2241	1000	600	7.877	25.473
7.50	2.4483	2000	1000	7.123	25.797
15.0	3.6724	3000	1600	3.288	16.868
30.0	7.3449	6000	4000		10.387

#### Physical and Chemical Characterizations after Modification

The surface chemical functional group of the membranes was observed by Fourier-transform infrared spectroscopy (FTIR) (Jasco, FT/IR-4700). The FTIR spectra were observed from an average of 64 scans in the wavenumber range of 3500–1000 cm^–1^ and a resolution of 4 cm^–1^. The surface wettability was evaluated by measuring the water contact angle formed when a water droplet is dropped on the PP fibrous membranes. This was performed with the aid of an optical contact angle measuring device (DataPhysics, OCA 15EC). In the experiment, a fixed volume of 4 μL of deionized water was slowly lowered onto the surface of the PP fibrous membrane, and then the contact angle of the water droplet with the membrane was measured using the software for the instrument. The change in the water contact angle was also observed in a span of 10 min to see whether the membranes would be capable of letting water pass through them.

The coating density of the membranes were measured using an analytical microbalance (Mettler Toledo, XP105 DeltaRange). Before the coating process, the dry weight of the PP membranes was measured and recorded as *W*_i_. After the drying process of the modification of the PP membranes, the membranes were weighed again and are recorded as *W*_f_. The coating density of the modified PP fibrous membrane was then calculated using the following formula
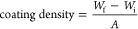
where *A* represents the surface area of the PP fibrous membrane. Three independent experiments in triplicate were measured and averaged. The error bars in the figures are the standard deviation of the measured results.

The surface and cross-sectional morphologies of the membranes before and after the coating process were visualized using a field emission scanning electron microscope (Hitachi S-4800). The membranes were cut into smaller pieces and then mounted on a stainless steel SEM stage by sticking them on carbon tape. For the cross sections of the membranes, they were trimmed while immersed under liquid nitrogen to prevent deformation and maintain the structural integrity of the cross section.

#### Biofouling Tests

Bacterial fouling tests were performed using an *Escherichia coli* containing a vector encoding a green fluorescent protein (GFP *E. coli*) to assess the bacterial adhesion on the modified PP membranes. GFP *E. coli* was first cultured by preparing its culture medium. Briefly, 200 mL of LB broth (Miller) was prepared and steam sterilized in an autoclave. The medium was further supplemented with 200 μL of ampicillin sodium salt resulting in a final concentration of 100 μg/mL. Then, 60 mL of the medium and 0.6 mL of a previously frozen GFP *E. coli* suspension having 10^7^ cells/mL were added to a 75T flask and allowed to incubate for 1 h. Afterward, 0.24 mL of isopropyl β-d-1-thiogalactopyranoside was added to induce the protein expression of GFP in the bacterial culture in the flask, which was then placed in a shaking incubator and was further incubated for 12 h. Simultaneously, PP membrane samples having a diameter of 1.3 cm were placed in a 24-well plate and 1 mL of sterile PBS was added to let the membranes swell and let them acclimatize, which were then incubated at 37 °C for 12 h. In order to initiate the bacterial adhesion test, the PBS in the well plate was replaced with 1 mL of the 12 h cultured bacterial solution, and the plate was placed in a shaking incubator for a total duration of 24 h. Within this 24 h adhesion time, the bacterial solution was replaced after 12 h with a fresh 12 h cultured bacterial solution. After 24 h of adhesion had passed, the bacterial solution was removed, and the samples were washed three times with sterile PBS before their observation under a microscope.

The fouling test with blood cells was conducted by using human whole blood. Samples were cut in 1.3 cm diameter membranes and were placed in a 24-well plate immersed in 1 mL of sterile PBS at 37 °C for 12 h for acclimatization. Afterward, the PBS was removed, and 1 mL of whole blood was added to the samples, which were then incubated in a shaking incubator at 37 °C and shaken at 120 rpm for 1 h to allow sufficient time of contact between the blood and the PP fibrous membranes. Subsequently, 1 mL of whole blood was removed, and the samples were washed with sterile PBS sufficient times to remove excess blood and weakly attached blood cells. Samples were then transferred to a new 24-well plate, and 1 mL of 2.5% of glutaraldehyde in PBS was added to each sample in order to fix the cells in the membrane. The well plate was then incubated at 37 °C for 60 min while covered with aluminum foil to minimize light exposure.

The biofouling test samples for both bacteria and blood cells were observed by using confocal laser scanning microscopy (CLSM) (Nikon A1R^+^). The data obtained from this test were analyzed quantitatively using the computer software, ImageJ. The analysis was performed with five replicates to ensure the accuracy and reliability of the results. Each sample was photographed 3 times at different locations. The average of the fouling coverage (in terms of %area) of a total of 15 images were calculated. Then, the fouling coverage for the unmodified PP membrane was set to 100% for an easier comparison with the rest of the samples. The standard deviation of these measurements was also presented as error bars in the figures. To determine statistical significance, a two-sample, two-tailed *t*-test was performed to compare each group with the untreated (virgin) control group. A *p*-value of less than 0.05 was considered statistically significant, indicating a significant difference between the means of the two groups being compared.

## Results and Discussion

### Surface Morphology of the Polypropylene Fibrous Membranes

**Figure 1:**
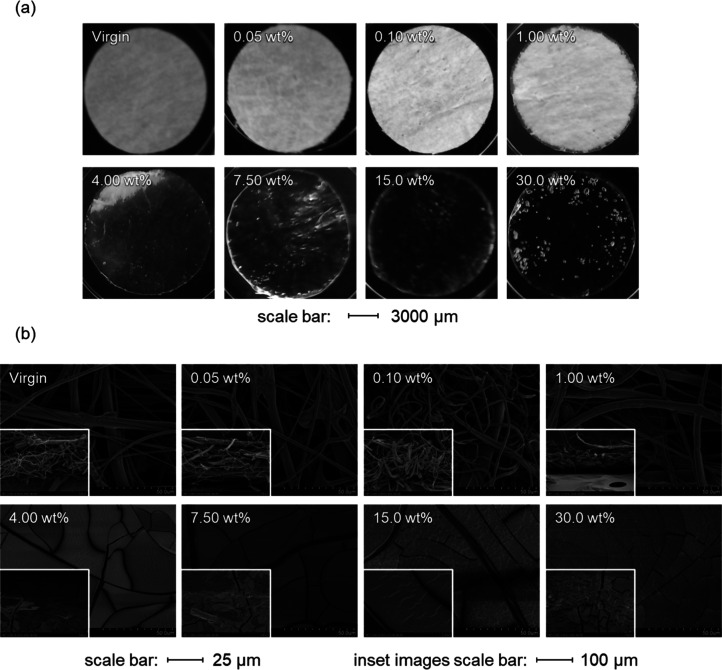
(a) Photograph of the PP membranes before and after the modification process. (b) SEM micrographs of the surface of the PP fibrous membranes before and after modification. The inset images are cross-sectional images of the membranes.

After the modification process for the PP fibrous membranes, they were photographed and examined under an electron microscope as presented in Figure 1. As seen from the photographs, the physical appearance of the membranes drastically changed when the solid concentration of reaction is above 1.00 wt % in the modification solution. The membranes become clear in appearance and seem to become a dense film. Upon observation under the electron microscope, it was confirmed that at solid content higher than 1.00 wt %, the PP fibrous membranes lost their original fiber structure and became denser. Membranes having solid contents of 0.05, 0.10, and 1.00% have shown to still keep their fiber structure. However, it can also be observed from the micrographs that there are some polymeric aggregates present. Similar observations were made where poly(sulfobetaine methacrylate) (poly(SBMA)) aggregates were present during the grafting of poly(SBMA) to PP membranes using plasma-induced modifications.^19^

### Chemical Characteristics of Polypropylene Fibrous Membrane Surface before and after Modification

**Figure 2:**
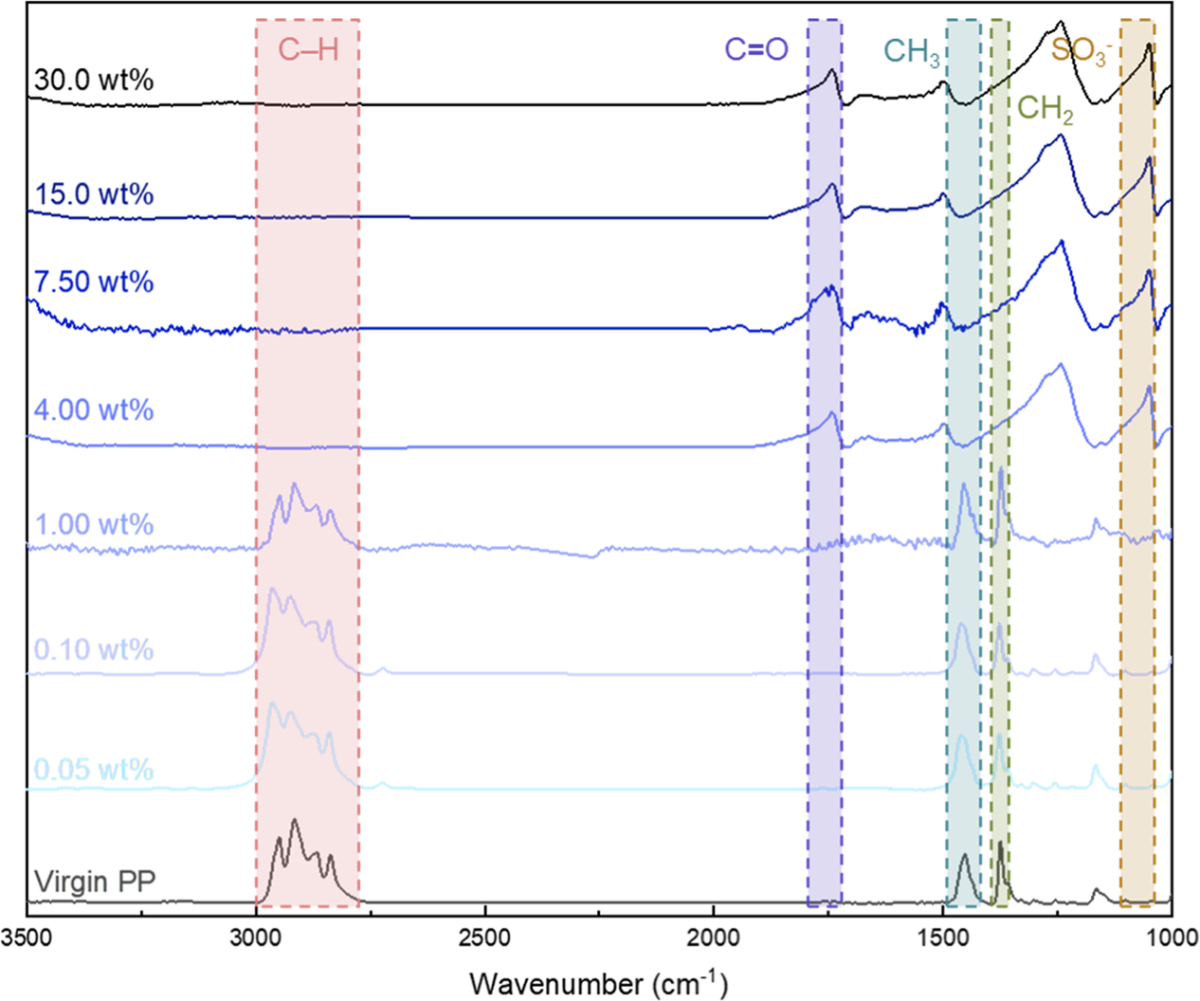
FTIR spectra of the PP membrane modified with SBMA via an interpenetrating process.

From the FTIR spectra of the samples, similar to the SEM results, a significant change can also be observed when the solid content in the reaction solution is higher than 1.00 wt %. In the case of the PP fibrous membrane surface, characteristic peaks were observed at wave numbers of 3000–2800 cm^–1^ corresponding to C–H bonds, 1460 cm^–1^ corresponding to CH_3_ bonds, and 1378 cm^–1^ corresponding to CH_2_ bonds.^27,28^ However, after undergoing modification, new characteristic peaks were detected at wave numbers of 1725 cm^–1^ corresponding to C=O functional groups and 1201–1043 cm^–1^ corresponding to SO_3_^–^ bonds,^29,30^ as shown in Figure 2. Under conditions where the solid content of the PP fibrous membrane was 0.05, 0.1, and 1 wt %, there was a slight decrease in the signal intensity associated with the PP fibrous membrane. This suggests that another layer is present on the PP membrane. However, the peaks of poly(SBMA) could not be observed on samples below 4.00 wt % which may signify that only a very thin layer of the poly(SBMA) is present in these conditions, which is not detectable in FTIR since the SBMA signal is negligible compared to the bulk PP signal. Conversely, no signals from the PP fibrous membrane were detected at solid contents of 4.00, 7.50, 15.0, and 30.0 wt %, while signals from the poly(SBMA) were observed.

**Figure 3:**
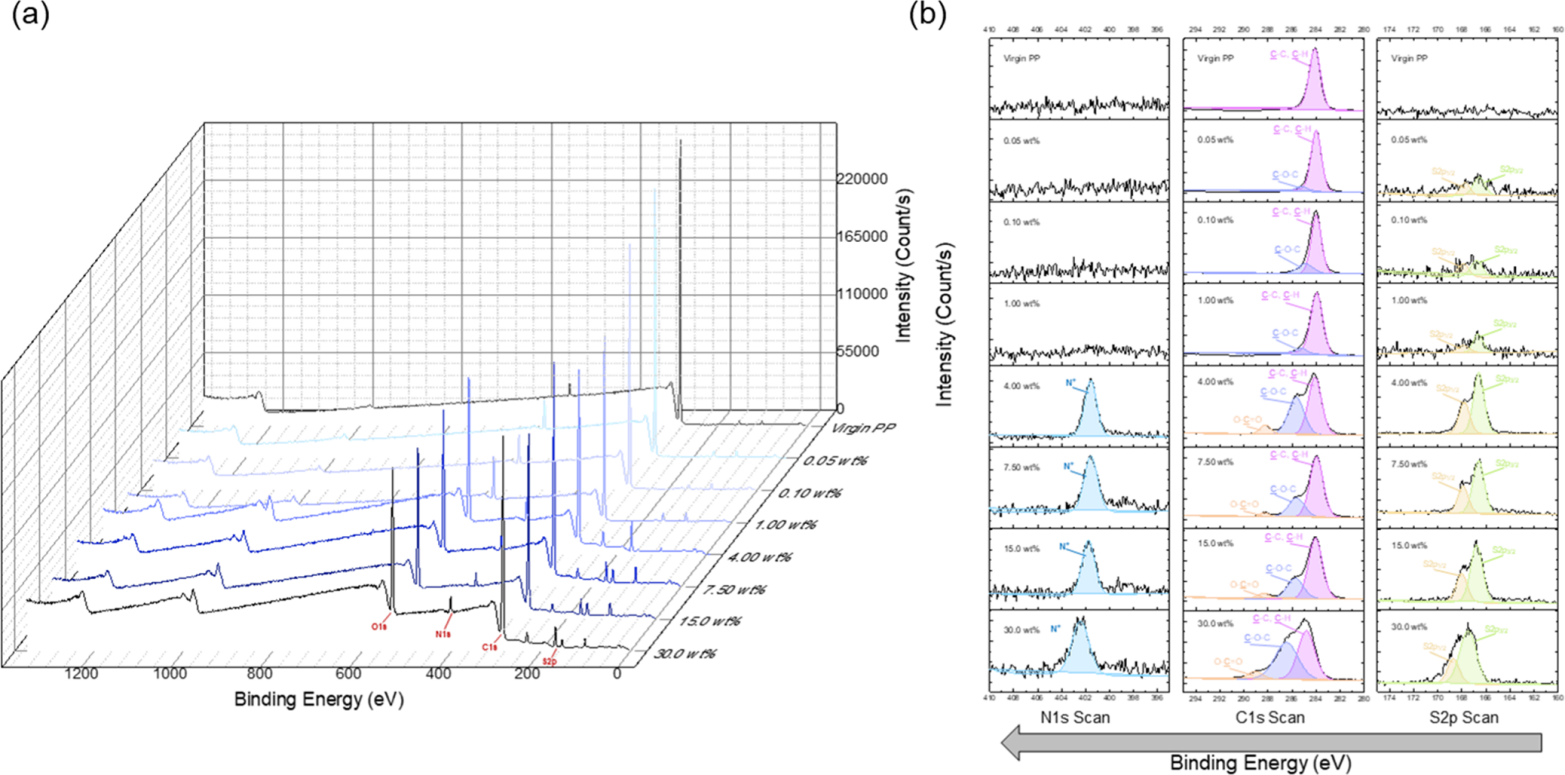
XPS spectra for the modified PP membranes. (a) Survey scan that can provide elemental analysis for the membranes. (b) Narrow elemental scans which can provide specific chemical bonding on the surfaces.

Since the presence of poly(SBMA) in the samples had lower than 4.00 wt % solid content in the reaction solution, the membranes were analyzed with X-ray photoelectron spectroscopy (XPS) as presented in Figure 3. From the XPS results, the unmodified PP membrane only shows a single peak from its C 1s core-level spectra corresponding to C–C or C–H bonding species in the polymer. For the modified PP membranes on the other hand, the deconvolution of the S 2p core-level spectra shows two distinct peaks corresponding to the S 2p_1/2_ and S 2p_3/2_ orbitals present in the SO_3_^–^ group from the poly(SBMA).^31^ However, similar to the FTIR spectra, the peaks for the PP membranes modified with lower than 4.00 wt % solid content of the reaction solution did not show any peaks from their N 1s core-level spectra. Alternatively, for PP membranes modified with the 4.00 wt % solid content of the reaction solution and above, a distinct peak is observed at 402 eV, which corresponds to the quaternary amine group in the poly(SBMA) structure.^32^ For the deconvolutions of the C 1s core-level spectra of the modified membranes, aside from the C–C and C–H peaks, a C–O–C peak is also observed at 286 eV, which comes from the poly(SBMA) structure. Additionally, for samples modified with 4.00 wt % and higher reaction solution, an additional O–C=O bonding is observed at 288 eV also from the structure of poly(SBMA). Also, presented in Figure 3a is the survey scan in the XPS analysis, which shows elemental composition of the PP membranes, which are also tabulated in Table 2. Aside from having the original peaks from PP, it can be observed that the modified PP membranes also have sulfur and nitrogen contents and distinct peaks of poly(SBMA). This signifies that there are two distinct polymer networks in the modified membrane, which are not entangled molecularly but in bulk.

**Table 2:** Elemental Composition of Each of the PP Membranes from Their XPS Survey Spectra

sample ID	atomic concentration (%)			
	C 1s	O 1s	S 2p	N 1s
virgin PP	97.64	2.36	*n.a.*	*n.a.*
0.05 wt %	93.72	5.6	0.32	0.37
0.10 wt %	93.74	5.58	0.23	0.46
1.00 wt %	89.72	9.62	0.4	0.26
4.00 wt %	67.78	21.9	5.47	4.84
7.50 wt %	72.91	19.71	3.81	3.58
15.0 wt %	71.01	22.77	3.39	2.82
30.0 wt %	72.19	20.77	3.7	3.35

### Surface Hydrophilicity and Coating Density on the PP Fibrous Membrane Surface

**Figure 4:**
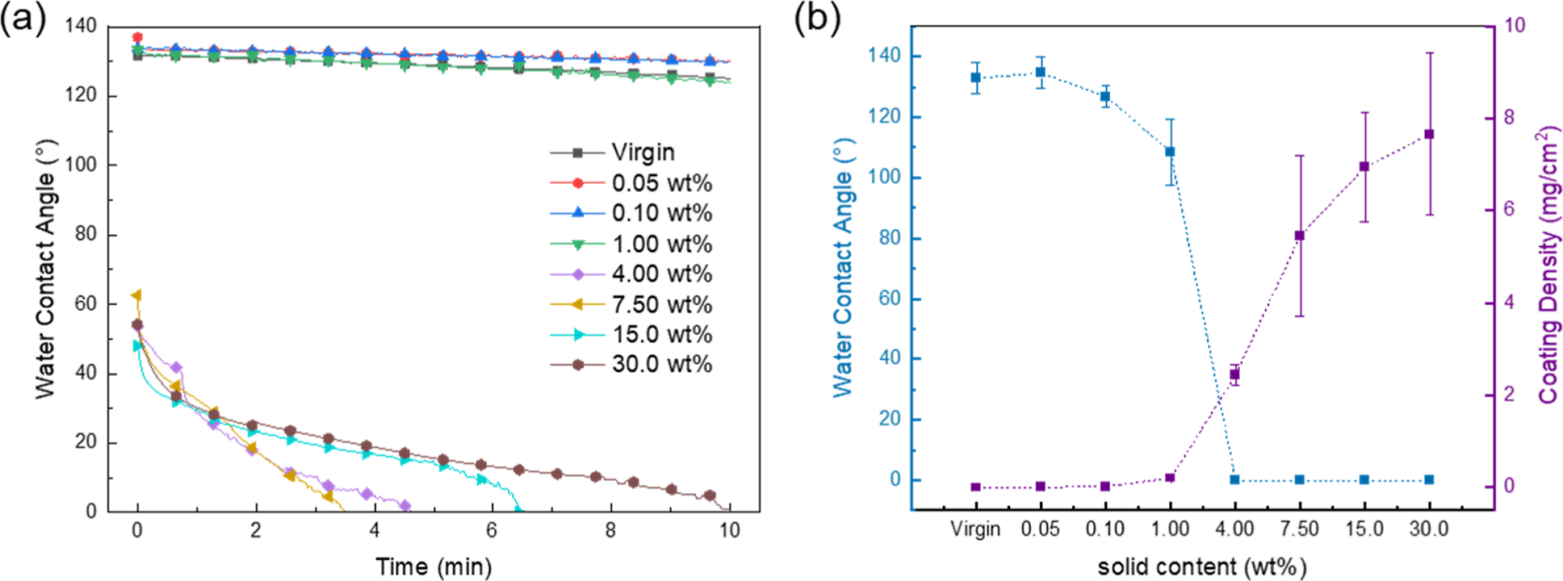
(a) Dynamic water contact angle of different solid content solutions. (b) Coating density and water contact angle in different reaction solution concentrations.

A change in hydrophilicity indicates the presence of altered surface chemistry on the membranes. The water contact angle of the unmodified PP fibrous membrane was measured at 132.97°, confirming its hydrophobic nature. Figure 4a shows a comparison of the water contact angles on the PP fibrous membrane after zwitterionization via the formation of a dual network at different solid contents. It can be observed that the water contact angles on the PP fibrous membrane coated with the poly(SBMA), which forms an entangled network, were significantly reduced for samples having solid contents of 4.00, 7.50, and 30.0 wt %. These contact angles approached nearly 0°, which demonstrates the effectiveness of the poly(SBMA) to provide excellent hydrophilicity. In the dynamic water contact angle measurement over a period of 10 min, there was a trend suggesting that an increase in solid content reduced the time for the water contact angle to decrease. For membranes with lower solid contents (0.05, 0.10, and 1.00 wt %), the reduction in water contact angle was less pronounced compared to those with higher solid contents, likely due to limited copolymer formation. Nevertheless, the contact angles of the 0.10 and 1.00 wt % samples were still lower than the unmodified PP membrane, measuring 126.87° and 108.47°, respectively—representing decreases of 6.10° and 24.50°. This indicates that even at lower solid contents, the zwitterionic modification of the membrane surface was successful.

Control over the molecular weight range and polymer chain length is also crucial for achieving excellent biocompatibility on zwitterionic interfaces.^33,34^ This control is influenced not only by the chemical properties of the anchoring units but also by other coating or reaction parameters. Figure 4b shows the 10th minute of water contact angle measurement, and the samples with solid contents of 4.00, 7.50, 15.0, and 30.0 wt % exhibited complete wetting behavior, with contact angles reaching 0°. Furthermore, an increasing trend in coating density was observed with higher solid contents. The coating densities for the 4.00, 7.50, 15.0, and 30.0 wt % samples were 2.436, 5.462, 6.943, and 7.664 mg/cm^2^, respectively—indicating substantial polymer deposition. It is important to note that at these higher solid contents, the variability in coating density also increased, as reflected by the larger error bars. This also coincides with the SEM images where it was seen that for these samples the original fiber structure of the PP membrane is already lost. Thus, at solid content higher than 4.00 wt %, we could see that the coating process is already dominated by aggregation of the poly(SBMA) on the PP membrane. These aggregates are also not stable as seen on the stability test presented in Figure S1 in the Supporting Information. It was evident that for samples with a solid content of 4.00 wt % or higher, weight loss was significant after immersion of the membranes in water for 3 weeks. On the other hand, for the solid contents of 0.05, 0.10, and 1.00 wt %, their coating densities are 0.004, 0.23, and 0.205 mg/cm^2^ respectively. Despite the reduced coating density, the 1.00 wt % sample, with a coating density of 0.205 mg/cm^2^, could still potentially provide antifouling properties. This is comparable to other materials, such as SAMs, which exhibit considerably lower coating densities but still demonstrate effective antifouling behavior.^35^

Additionally, to emphasize the effect of the poly(SBMA) aggregates on conditions with a higher solid content, the permeability of the membranes was evaluated. The unmodified membrane together with the membranes modified with 1.00 and 7.50 wt % were evaluated. It can be seen in Figure S2 in the Supporting Information that the flux of the membrane modified with a solid content of SBMA of 1.00 wt % enhanced the pure water flux while the one modified with 7.50 wt % was reduced. Coinciding with the SEM images and the weight loss data, it can be ascertained that the polymer aggregates formed in membranes modified with SBMA solid content higher than 1.00 wt % affect the membrane performance negatively.

### Bacterial Adhesion Capacity

**Figure 5:**
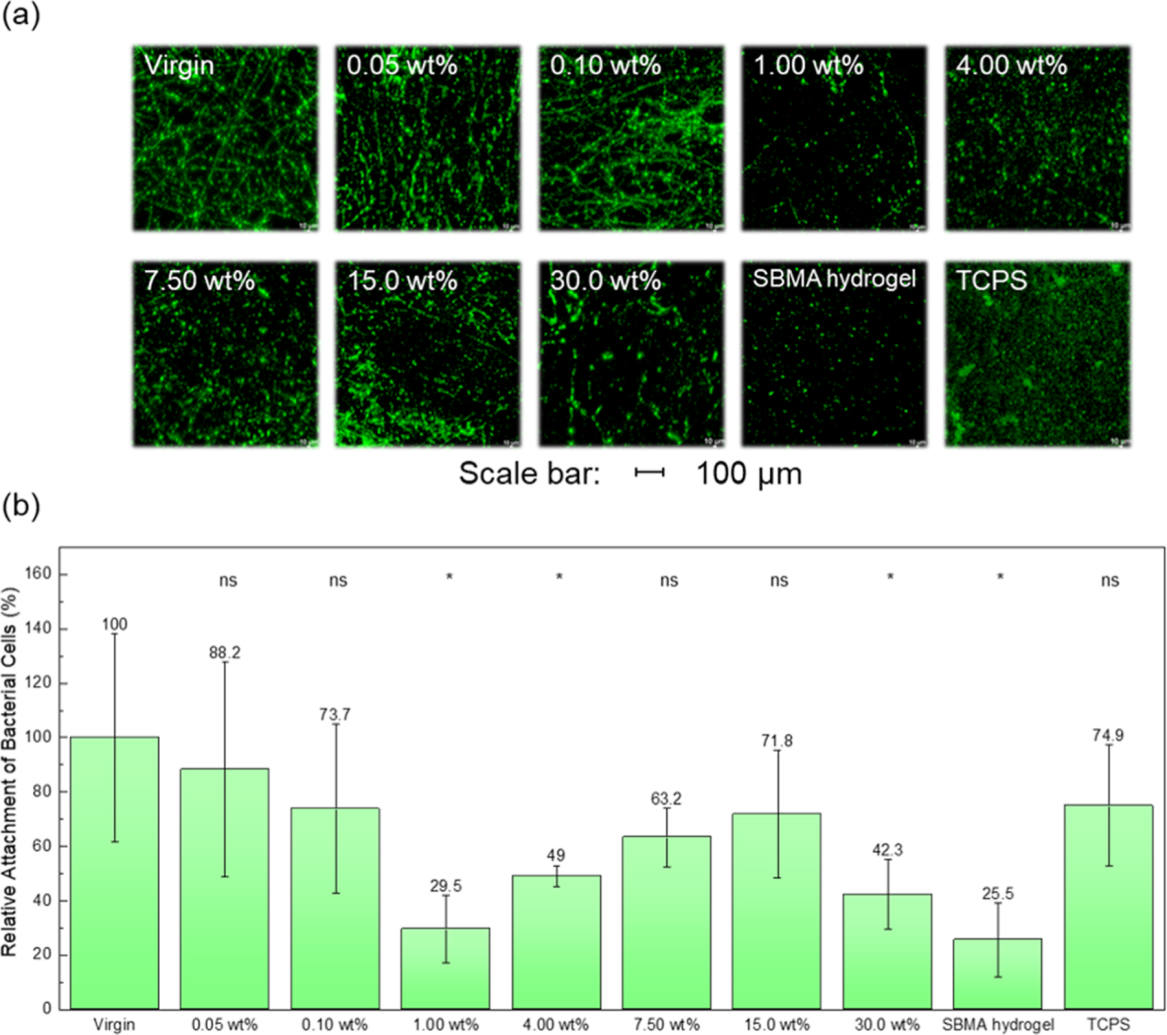
GFP *E. coli* attachment of zwitterionic PP fibrous membrane. (a) Confocal micrographs and (b) quantified bacterial attachment via ImageJ together with the statistical significance of each condition when compared to the Virgin sample (ns—no significant difference; *—*p*-value <0.05).

*E. coli* is a common bacterium present in water and the intestines of warm-blooded organisms.^36,37^ Due to its deformability, this Gram-negative bacterium can penetrate very small pores and establish hydrophobic interactions with membrane materials.^38^ Both structurally and chemically mediated interactions contribute to irreversible contamination. Furthermore, research has shown a correlation between bacterial adhesion and protein adsorption on material surfaces, making bacterial attachment a good indicator of antifouling performance.^39^ In this experiment, GFP *E. coli* was used to assess bacterial adhesion. Figure 5 presents the images and corresponding quantitative data obtained from CLSM. The results indicate that the unmodified PP fibrous membrane exhibited the highest level of bacterial adhesion, which can be attributed to its hydrophobic nature that promotes interactions with the bacteria. This can be attributed to the hydrophobic nature of the PP fibrous membrane, which promotes interactions with the bacteria. Notably, the membranes modified with 1.00 and 4.00 wt % solid content demonstrated the most effective resistance to bacterial attachment. Lower solid content samples exhibited higher bacterial adhesion, suggesting an insufficient poly(SBMA) content to provide adequate antifouling properties. Conversely, higher solid content samples that also exhibited increased bacterial attachment can be attributed to an excessive amount of poly(SBMA) on the surface, which may become softer when hydrated, facilitating the physical entrapment of bacteria. The membrane modified with 1.00 wt % condition showed the greatest reduction in bacterial attachment, being able to reduce a little more than 70% compared to the unmodified PP membrane. It is also important to note that the higher solid content of the SBMA shows increased fouling. Even though the ratio of the initiator and the monomer in each condition was kept constant, polymer aggregates were still observed from the SEM images of the membranes with a higher solid content. This may indicate higher molecular weights of poly(SBMA) in the higher solid contents. In the design of antifouling materials, the material chemistry is not the sole criteria. Different factors, such as packing density, chain length, and flexibility, also come into play. The molecular weight being too high would create gaps in the packing density.^40^ Also, aggregates of even zwitterionic polymers have been shown to aggravate the fouling experienced by membranes.^41^ In addition, the hydration capacity of the membranes was also evaluated. There are more interchain interactions among the SBMA groups in the aggregated poly(SBMA) in the membranes and thus reducing the bound water formed in the hydration layer. As seen in Figure S3, the membrane modified with 1.00 wt % SBMA had the highest hydration capacity. The hydration capacities of membranes with polymer aggregates were seen to be less or negative. This is also due to the instability of the polymer aggregates. The aggregates get dissolved in water, and thus the weight of the membranes after immersion in water was lower compared to the initial weight of the membranes.

### Surface Blood Adhesion Capability

**Figure 6:**
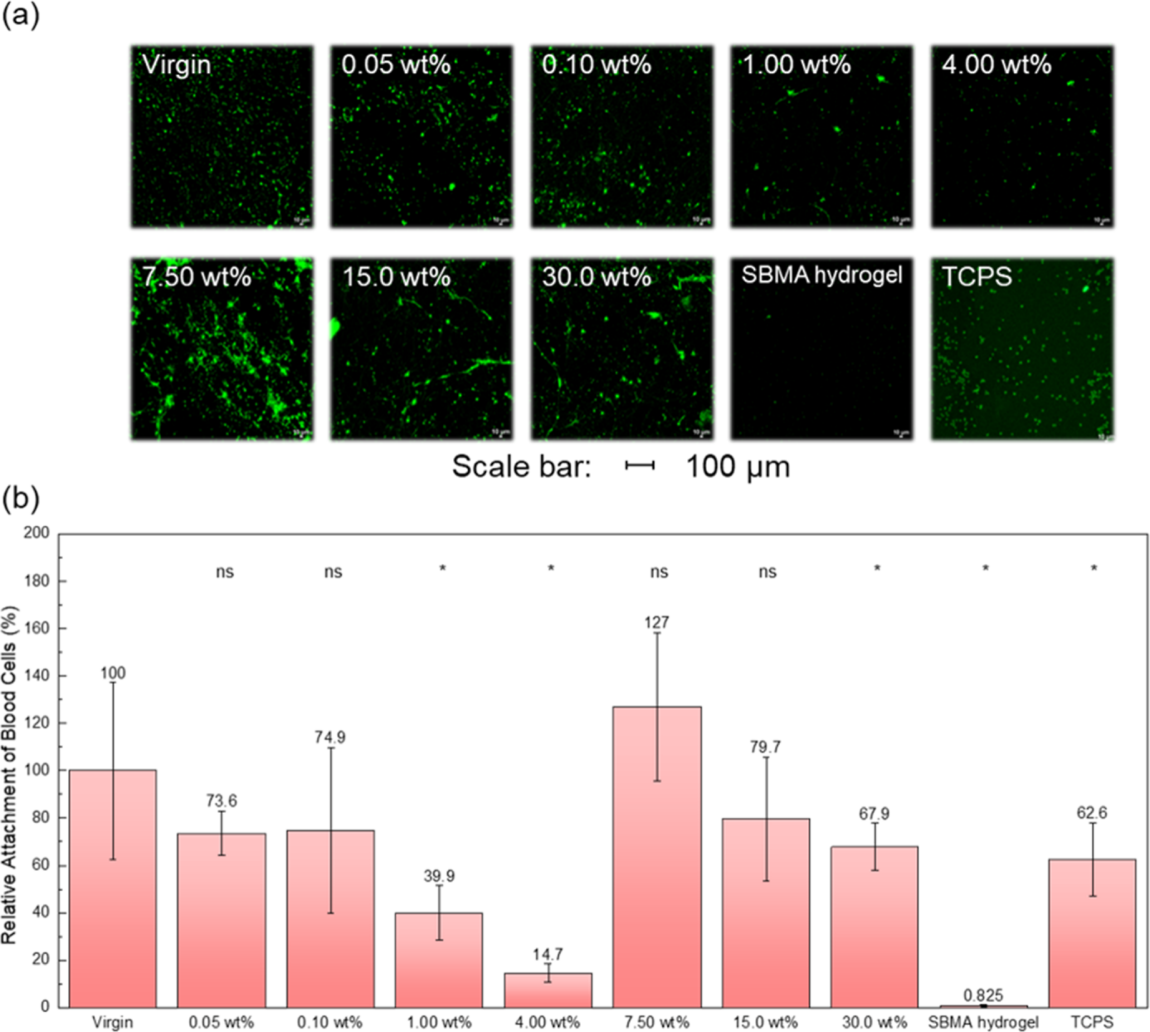
Blood cell attachment experiment on zwitterionic PP fibrous membranes. (a) Confocal micrographs and (b) corresponding quantified blood cell attachment via ImageJ together with the statistical significance of each condition when compared to the Virgin sample (ns—no significant difference; *—*p*-value <0.05).

The interaction of blood with biomaterials can initiate a cascade of events, beginning with the adsorption of plasma proteins and clotting factors onto the material surface. This can lead to the subsequent attachment of larger biomolecules, such as blood cells, ultimately resulting in thrombus formation, immune reactions, and other complications.^42,43^ Therefore, a key requirement for a biocompatible or antifouling material is the minimization of blood cell attachment. In this study, blood cell adhesion was evaluated by exposing the modified membranes to human whole blood. Figure 6 shows the CLSM images and corresponding quantitative data for the blood cell attachment experiment. Consistent with the bacterial attachment results, the unmodified PP fibrous membrane, with its inherent hydrophobicity, exhibited the highest degree of blood cell attachment, indicating a strong interaction between the hydrophobic surface and the blood cells. It is also evident that the surface of the PP fibrous membrane modified through the in situ polymerization of poly(SBMA) showed reduced blood cell adhesion. Among the various solid content conditions, the 4.00 wt % concentration exhibited the most pronounced antifouling properties, reducing blood cell adhesion by 85% compared to the unmodified membrane. The 1.00 wt % solid content modification also significantly reduced blood cell adhesion, achieving a 60% reduction relative to the unmodified PP membrane. In contrast, the remaining solid content conditions yielded a more moderate reduction in blood cell adhesion, ranging from 30% to 40% compared with the unmodified PP membrane.

## Conclusions

In this study, polymer poly(SBMA) was utilized to form a dual network with the PP fibrous polymeric membrane via in situ polymerization. The zwitterionic nature of poly(SBMA) facilitates the formation of a hydration layer on the inherently hydrophobic PP surface, thereby enhancing biocompatibility. While PP membranes modified with both 1.00 and 4.00 wt % poly(SBMA) demonstrated excellent antifouling properties against bacteria and blood cells, the 4.00 wt % modification resulted in a disruption of the fibrous structure of the PP membrane. Consequently, the optimal modification was achieved with 1.00 wt % poly(SBMA), which enhanced hydrophilicity while preserving the structural integrity of the membrane. Despite the minimal reduction in water contact angle, the optimized membrane significantly reduced bacterial adhesion by 70% and blood cell adhesion by 60%. Importantly, this improvement in biocompatibility was achieved without the incorporation of hydrophobic segments within the modifying polymer. The formation of a dual network between poly(SBMA) and the PP fibers enabled a stable modification, simplifying the conventional approaches to modifying hydrophobic materials and offering potential for scalable coating processes. This advancement holds promise for applications in diverse fields, including blood purification, food processing, and microbial filtration.

## Supporting Information Available

Stability via weight loss, permeability (pure water flux), and the hydration capacity of the membranes (PDF)
