# Supplementary material for: Bioinert Fibrous Polypropylene Membranes via In Situ Polymerization of Zwitterionic Poly(sulfobetaine methacrylate)
Source: Langmuir. 2025 Feb 10;41(7):4524–32. doi: 10.1021/acs.langmuir.4c04226 (PMC11866916; doi:10.1021/acs.langmuir.4c04226)
Supplement: Supplementary file 1 — la4c04226_si_001.pdf [file la4c04226_si_001.pdf]

Supporting Information

Bioinert fibrous polypropylene membranes via *in-situ* polymerization of zwitterionic poly(sulfobetaine methacrylate)

Gian Vincent Canlas Dizon, [†] *Chiao-Ling Chang,* [†] *Chih-Chen Yeh,* [†] *Chung-Jung Chou,* [†] *Jheng-Fong Jhong,* [‡] *Jie Zheng,* [§] *and Yung Chang**, [†]

[†]R&D Center for Membrane Technology and Department of Chemical Engineering, Chung Yuan Christian University, 200 Chung Pei Rd, Taoyuan 32023, Taiwan

[‡]PuriBlood Medical, Baoshan Township, Hsinchu County 300096, Taiwan

[§]Department of Chemical, Biomolecular, and Corrosion Engineering, The University of Akron, Akron, Ohio 44325, USA

*ychang@cycu.edu.tw

Number of pages: 4

Number of figures: 3

Figure S1. Weight loss of the membranes after immersion in DI water for 3 weeks. S2
Figure S2. The water permeability of the membranes evaluated at 1 atm for 30 minutes........... S3
Figure S3. Hydration capacity of the modified PP membranes. S4

Supporting Information

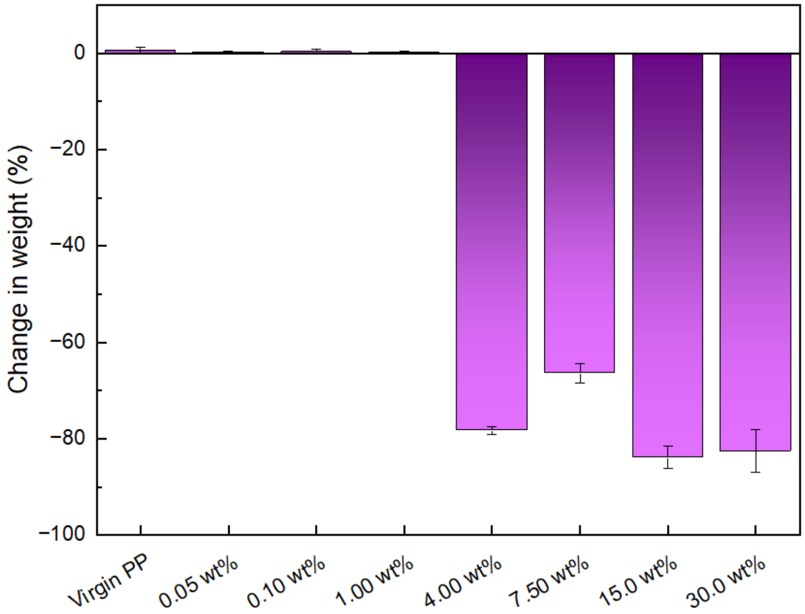

Figure S1. Weight loss of the membranes after immersion in DI water for 3 weeks.

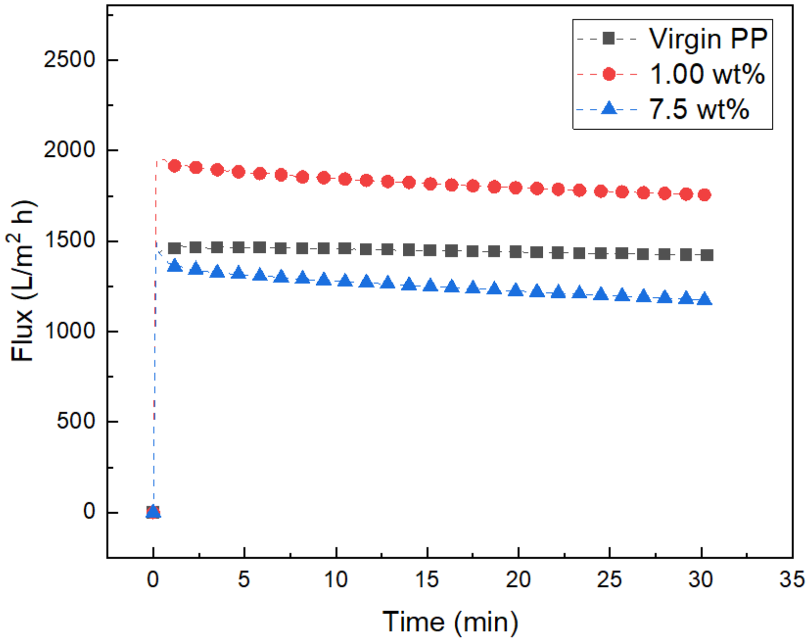

Figure S2. The water permeability of the membranes evaluated at 1 atm for 30 minutes.

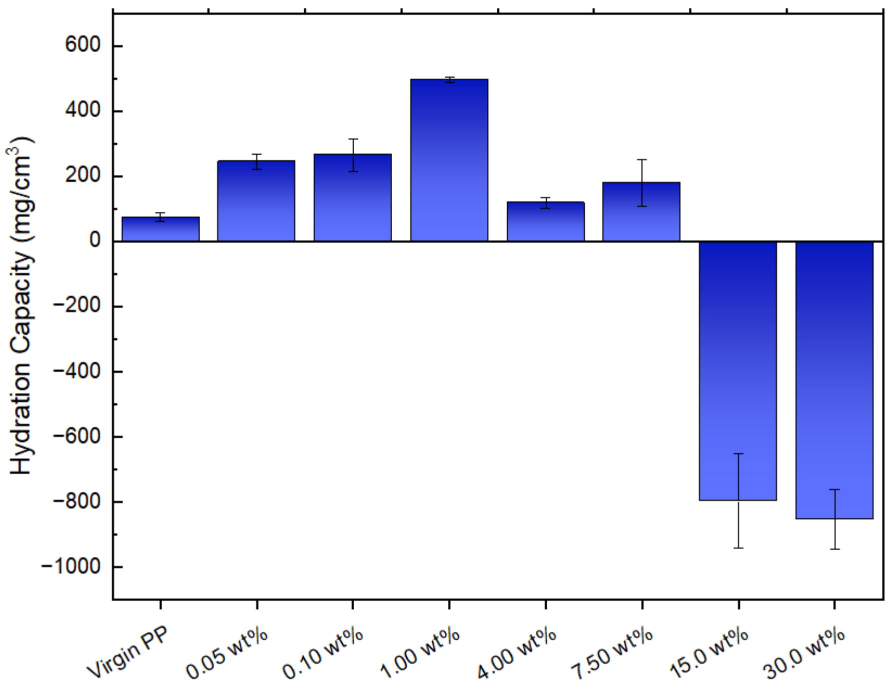

Figure S3. Hydration capacity of the modified PP membranes.
